# Measuring cognitive load of digital interface combining event-related potential and BubbleView

**DOI:** 10.1186/s40708-023-00187-7

**Published:** 2023-03-03

**Authors:** Shaoyu Wei, Ruiling Zheng, Rui Li, Minghui Shi, Junsong Zhang

**Affiliations:** 1 Department of Artificial Intelligence, Xiamen University, Xiamen, 361005 Fujian China; 2 National Engineering Laboratory for Educational Big Data, Central China Normal University, Wuhan, 430079 Hubei China

**Keywords:** Helmet mounted display systems, Event-related potential, Graphical user interface, Cognitive load, Eye tracking

## Abstract

Helmet mounted display systems (HMDs) are high-performance display devices for modern aircraft. We propose a novel method combining event-related potentials (ERPs) and BubbleView to measure cognitive load under different HMD interfaces. The distribution of the subjects’ attention resources is reflected by analyzing the BubbleView, and the input of the subjects’ attention resources on the interface is reflected by analyzing the ERP’s P3b and P2 components. The results showed that the HMD interface with more symmetry and a simple layout had less cognitive load, and subjects paid more attention to the upper portion of the interface. Combining the experimental data of ERP and BubbleView, we can obtain a more comprehensive, objective, and reliable HMD interface evaluation result. This approach has significant implications for the design of digital interfaces and can be utilized for the iterative evaluation of HMD interfaces.

## Introduction

Helmet mounted display systems integrate advanced electronic display and head tracking systems. Through microdisplay technology, information about the flight system and aircraft situation is projected directly in front of the pilot, allowing him to acquire what he needs at any time, thereby significantly improving the aircraft’s operational effectiveness [1–5]. A reasonable HMD interface improves the pilot’s awareness of the situation and ensures his accurate cognitive judgment under highly tense conditions. However, the HMD interface of modern aircraft brings convenience to pilots and also a vast amount of information. The pilot needs to prompt, capture, track and eventually lock onto the off-axis target through the HMD interface in flight. The pilot must quickly and accurately extract information from the HMD interfaces during this process. If the “symbol system” of the mask on the helmet is difficult to recognize, the helmet will be unable to fully meet the requirements of the aircraft, resulting in an unmanageable cognitive load for the pilot and causing the pilot to be unable to work properly. Therefore, evaluating and reducing the cognitive load of pilot caused by HMD interface is very important to achieve normal adequate operation efficiently in flight [2].

Eye tracking records the distribution process of the user’s interaction attention to evaluate the graphical interface [6, 7]. Eye tracking refers to the tracking of eye movement by measuring the position of the eye’s gaze point or the eye’s movement relative to the head. Eye tracking is regarded as an effective HMD interface evaluation technique because it can correctly depict how visual channels are processed in time and space [8]. However, eye tracking experiments cannot be used outside the lab since professional eye tracking equipment is pricey and requires testing prior to the experiment (e.g., EyeLink, ISCAN) [9]. BubbleView replaces eye tracking with mouse clicks and is not limited by the experimental environment. In addition, BubbleView can simulate the blurred visual edge and a constrained tiny area of the human visual fovea [9], which can slow down users’ exploration of images and aid in our understanding of the cognitive rules of users. However, the brain will show apparent changes in the process of interface evaluation with further information processing. When evaluating graphical interfaces, eye tracking methods such as BubbleView cannot directly disclose the brain’s workings.

To this purpose, ERP [10, 11] can be used to investigate the internal workings of the user’s brain to assess the graphical interface. The electroencephalogram (EEG)-based research technique known as ERP is well-liked in various domains. Jia et al.[8] use ERP to investigate the brain electrophysiological characteristics induced by users in the process of interface cognition and evaluation of different similarities. It reveals the cognitive rules of users for the different graphic interfaces through attribute variations of the acquired brain physiological indicators. Eye tracking is less accurate in reflecting the brain’s internal workings than ERP technology. Combining ERP with eye tracking can explore the complete cognitive evaluation process from input to allocating attention resources.

In this work, we take the HMD interface as the specific research object and combine ERP and BubbleView to evaluate the HMD interface. Unlike previous work, which only uses one of the methods to evaluate the cognitive load under the interface, either with ERP or with eye tracking, making it impossible to evaluate cognitive load thoroughly. The input of the subjects’ attention resources on the interface is revealed by analysis of the ERP’s P3b and P2 components. The distribution of the subjects’ attention resources is reflected by recording the number of mouse clicks in BubbleView and analyzing the produced visual importance heatmap. This enables us to get a more thorough, impartial, and trustworthy evaluation of the HMD interface.

## Related work

Cognitive load theory was first proposed by John Sweller in 1988 [12], based on the early research of Miller et al. Cognitive load is closely related to the difficulty of cognitive tasks. Thus, the quality of digital interface can be evaluated by measuring the cognitive load of the interface [13–15]. Existing approaches to measuring cognitive load and evaluating digital interface mainly include eye tracking, ERP, etc. We mainly review the methods closely related to our research.

Eye tracking can demonstrate the area of users’ visual interest [16] and assist us in comprehending subjects’ eye gaze patterns and attentional allocation. Wu et al. [17] applied eye movement tracking technology to the usability evaluation of smartwatch interface and quantitatively analyzed the internal differences between interfaces. Açik et al. [18] evaluated two different surgical interfaces (SI) by recording the eye movements of doctors and participants without medical expertise during interaction with an SI that directs a simulated robotic cryoablation task. However, most eye tracking equipment is pricey and requires complicated calibration, making it challenging to use in large-scale investigations outside lab settings.

Using cameras on simple personal portable devices [19–21] can be a more economical eye tracking method. Although this method is simple and cost-effective, it has yet to be widely adopted because of the poor accuracy of eye tracking data, and the camera calibration and light conditions must be set before use. Therefore, there are cursor-based visual attention tracking methods making use of the connection between the eye fixation point and the cursor fall point, greatly simplifying the parameters that must be set during the data acquisition process and providing a viable alternative to traditional eye tracking studies [20, 22–25]. BubbleView is a cursor-based, window-moving method [9, 26], which replaces eye gaze by collecting the user’s mouse clicks on static images. BubbleView shows the user a blurred image and asks them to click it to expose a small circular bubble area [27, 28], that is, restore the blurred area to its original resolution. This purpose is to simulate human vision—the edge blur area in the fovea and the limited area where the fovea is in focus. BubbleView effectively slows down cognitive processing, since it takes longer for the user to move the mouse around the interface and decide where to click than for the human eye to glance at it. The click data of BubbleView are extremely close to human eye movement data when respondents were asked to describe information visualization images like icons and tables under the descriptive task. Combining the original image with the BubbleView mouse click data creates a “visual importance heatmap ”, which can describe the images’ salient regions that attract the most visual attention and use it to analyze HMD interface instead the eye movement heatmap. Similar to eye movement, we can also analyze the user’s visual perception and cognitive process through the mouse click data of BubbleView. The average time it takes to click the mouse will be shorter if the HMD interface is simpler to understand.

ERP reflects the cognitive processing of information [29–31], which can make up for the deficiency of eye tracking on reflecting information processing inside the brain.

P300 is one of the components of ERPs, which reaches its maximum amplitude about 300ms after the stimulus is presented. P300 is an essential indicator of task difficulty [32–34]. Since P300 component was discovered, research has shown that P300 component is composed of two sub components, P3a and P3b [35]. P3a is produced in response to the processing of sensory stimuli with frontal lobe activation from attention-driven working memory changes. P3b is produced as a result of temporal/parietal lobe activation from memory and context updating operations and subsequent memory storage, has somewhat longer latency, and is less sensitive to habituation than P3a [14]. To eliminate the habituation factor, we only adopted the P3b component rather than combining P3a and P3b to measure cognitive load in our experiment.

There are typically two different experimental methods that can produce P3b. The first is a dual-task paradigm, which examines how much of the individuals’ attention is still focused on the primary task. The research adopts a dual-task paradigm because vision and auditory share cognitive resources [36, 37]. Complex tasks consume much of the cognitive resource, reducing the amount of cognitive resource allocated to tasks other than the current one [38, 39]. So when subjects are under high cognitive load in primary tasks, they would be less responsive to additional stimuli of secondary tasks such as sound stimuli [40–42]. The other is the visual oddball paradigm, in which recognizing a target stimulus includes working memory, attention, stimulus evaluation and pattern matching [43]. A target stimulus significantly induces P3b. Instead of using the dual-task paradigm in our study, we directly adopted the visual oddball paradigm to test the attention resources devoted to the current activity. Therefore, the P3b component can intuitively measure the relationship between interface layout factors and cognitive load.

In addition, many researchers have found that some other ERP components correlate with task difficulty using the adaptive cognitive load paradigm [32, 44, 45]. For example, P2 is a crucial index to assess users’ cognitive load since it is sensitive to changes in cognitive load [33]. From the perspective of cognition, P2 reflects a pre-attention-alertness mechanism, which can improve the participants’ awareness of stimuli. The amplitude of P2 is directly related to the complexity of visual stimuli [46], indicating that P2 reflects a low level of cognitive difficulty information related to the physical properties of visual stimuli. The P3b component mainly reflects a high level of cognitive difficulty in information synthesizing high-level semantics. Combining P2 and P3b can more accurately measure users’ cognitive load from two different cognitive levels.

In ERP study, we studied the cognitive load of subjects from two different levels, namely low-level physical attribute meaning of stimulus and high-level semantic meaning of stimulus, and measured users’ cognitive load under different HMD interfaces by analyzing P2 and P3b components in combination with the oddball paradigm. If the HMD graphical user interface is harder to remember and recognize, the latency and amplitude of P2 and P3b will be longer and larger.

**Fig. 1:**
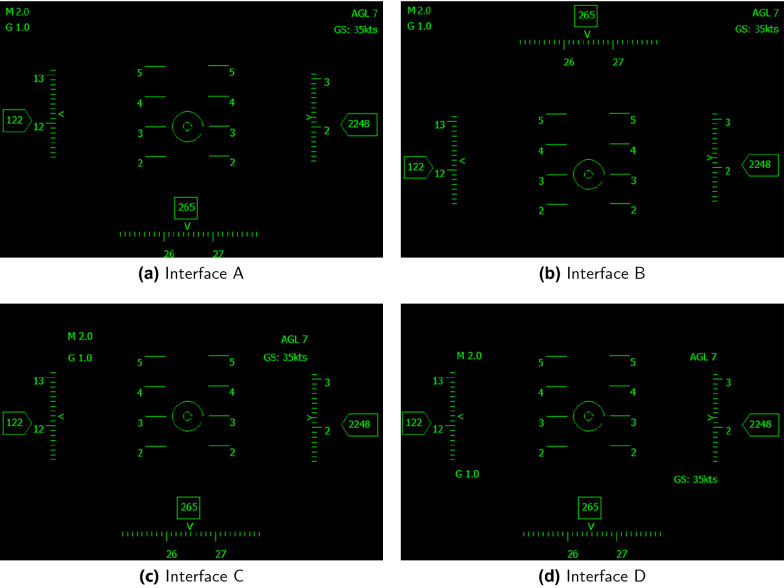
Combining the classical aircraft HMD interface with flight simulation software FlightGear, determined the flight parameters and shape of our HMD interface element and designed four different HMD interfaces. The only difference between interface **A**, **B** lies in the location of azimuth icon. The only difference between interface **C**, **D** is the location of icon “M 2.0”, “AGL 7”, “G 1.0”, “GS: 35kts

## Methods

### ERP experiment

#### Participant

A total of 19 undergraduates and postgraduates of Xiamen University were recruited to participate in the paid experiment (10 males and 9 females, ages 19–26 years, average age 22.6, standard deviation 2.2), all of them were right-handed, with normal naked eye vision or corrected normal vision. All signed an informed consent form.

#### Dual stimulus “oddball” task

The oddball paradigm is one of the commonly used paradigms in ERP experiments. It refers to the random presentation of two stimuli of the same sensory channel in an experiment, and the probability of occurrence of the two stimuli is very different. The high-probability stimulus is called the standard stimulus, which is the background of the whole experiment. The low-probability stimulus is called deviant stimulus. The probability of the deviant stimulus is around 20% and the probability of the standard stimulus is around 80%. If the subject is asked to respond to the deviant stimulus, the deviant stimulus becomes the target stimulus at this time.

**Fig. 2:**
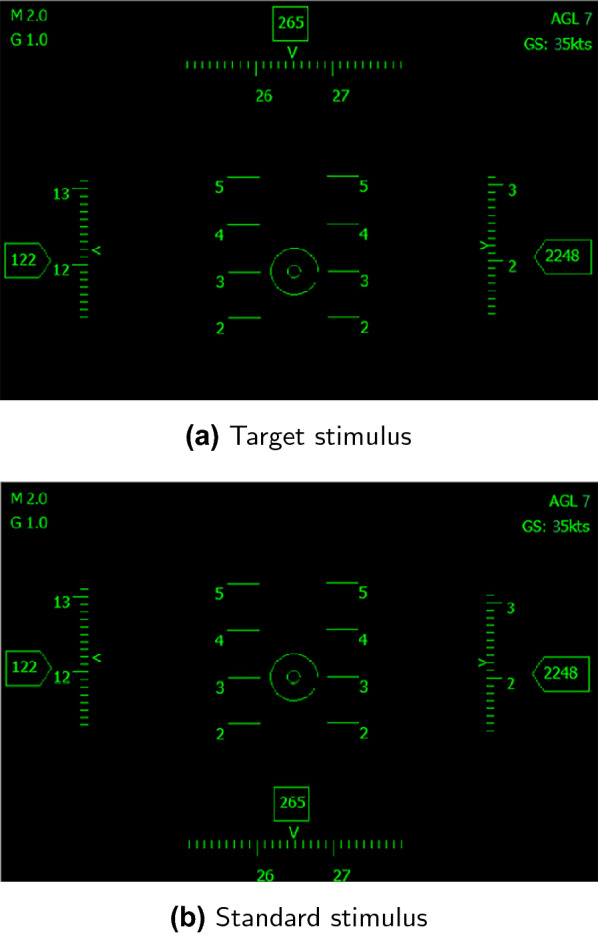
Indicating interface in the experiment. Target stimulus means that when subjects see the interface, they should click the left mouse button as soon as possible. Standard stimulus means that subjects do not have to react when they see the interface. The interface is only used as instruction at the beginning, and the corresponding text information will not appear during the experiment

In this work, the stimulus was the four different HMD interfaces shown in Fig. 1. The experiment was divided into four groups, and each participant completed all four groups of experiments.

At the beginning of the experiment, an indicating interface was presented (Fig. 2) which defines target stimulus and standard stimulus in each group. After participants confirmed that they remembered the difference between target stimulus and standard stimulus, they pressed the blank space key to enter the next step. The target stimulus and the standard stimulus will randomly appear in the middle of the screen. Each group of experimental target stimulus randomly appears 40 times (the probability is 0.2), the standard stimulus appears 160 times (the probability is 0.8), and the stimulus presentation time is 1000 ms. Subjects were asked to click the left mouse button as soon as they see the target stimulus. When the standard stimulus appeared, subjects will not need to make any response. From last stimulus disappear until next stimulus present, time interval length was 500ms. During this period, subjects were asked to gaze at the fixation cross presented in the middle of the screen.

Since the difference between the target stimulus and standard stimulus will directly affect P3b and P2 amplitude, each group of the experimental target stimulus and standard stimulus was different. To directly compare and analyze interface A and B, interface C and D, we exchanged the target and standard stimulus of the first and the third group, and set up the second and the fourth group of experimental. Table 1 shows the stimulus in each group.

The stimulus was presented in the center of the DELL P2314H LCD display on a black background with a refresh rate of 60 fps. We used psychology software E-prime 2.0 to control the presentation of the stimuli. The eyes of the subjects were fixed 70 cm away from the monitor. The horizontal and vertical angles of the stimulus relative to the subject’s field of view were both less than $6^{\circ }$.

**Table 1:** Standard stimulus and target stimulus in each group

	Standard stimulus	Target stimulus
First group	A	B
Second group	B	A
Third group	C	D
Fourth group	D	C

At the same time, there will be a rest after the completion of each group to avoid excessive fatigue of the subjects. The subjects must keep their head stable and close their eyes for rest. The subjects can define their own break time. There was a training stage before the formal experiment that aims to familiarize subjects with the task process. After the training stage, the formal experiment will begin and last about 25 min.

#### Procedure

The experiment was conducted in a closed, soundproof room with standard indoor lighting. Each subject sat on chair in front of the monitor, and the head was placed on the bracket of the 66vt-yt2b slit lamp table to maintain stability. Before the beginning of the experiment, the guide words appeared on the screen and the subjects were asked to read it carefully. They were asked to blink naturally during the experiment. After placing the electrodes and the researchers left, subjects pressed the space bar to begin the experiment.

#### EEG signals recording and preprocessing

Neuroscan 4.5 (http://www.neuroscan.com/) was used for data recording and analysis. A 64-channel EEG electrode cap of an international 10–20 system was used for data collecting. The ground electrode was FPz and the reference electrode was from the nose tip. Vertical eye movements were recorded with electrodes placed on the supraorbital and infraorbital ridges of the left eye. The horizontal eye movements were recorded with electrodes placed laterally to the outer canthi of both eyes. The impedance of all electrodes was kept lower than 5 $\hbox {k}\Omega$ during recording, and the sampling rate was 1000Hz. The EEG and electrooculogram (EOG) signals were amplified using a band-pass of 0.05–100Hz. After continuous EEG recording, the data were processed offline. Automatized artifact rejection was used to eliminate trials during which detectable eye movements, blinks, muscle potentials, or amplifier blocking occurred. Then, EEG waves were filtered for each subject with a band-pass Gaussian filter (0.15–30Hz) to reduce residual high-frequency artifacts in the waveform. We selected the period from 200 ms before the appearance of target stimulus to 1000 ms after the appearance of target stimulus as the EEG segmentation time. Trails in which the EOG or EEG exceeded $\pm 100\mu \hbox {v}$ were excluded.

### BubbleView experiment

#### Participant

As described by Kim et al. [9], participants about 15 people are enough to obtain a good result for descriptive BubbleView experiment. 20 participants took part in the study (including 9 males and 11 females, aged 19 to 26 years, with an average age of 24, the standard deviation of 2.1), all were right-handed, naked eye eyesight or corrected vision was normal, and no subjects had psychological disorders.

#### Mouse clicks and picture description task

In this task, four pictures were presented in random order. The experiment required users to reveal the picture by clicking and describe the picture as thoroughly as possible during the exploration of the picture. There was no time limit for this task. The blur radius was set to 10 pixels, the bubble radius was set to 30 pixels.

**Fig. 3:**
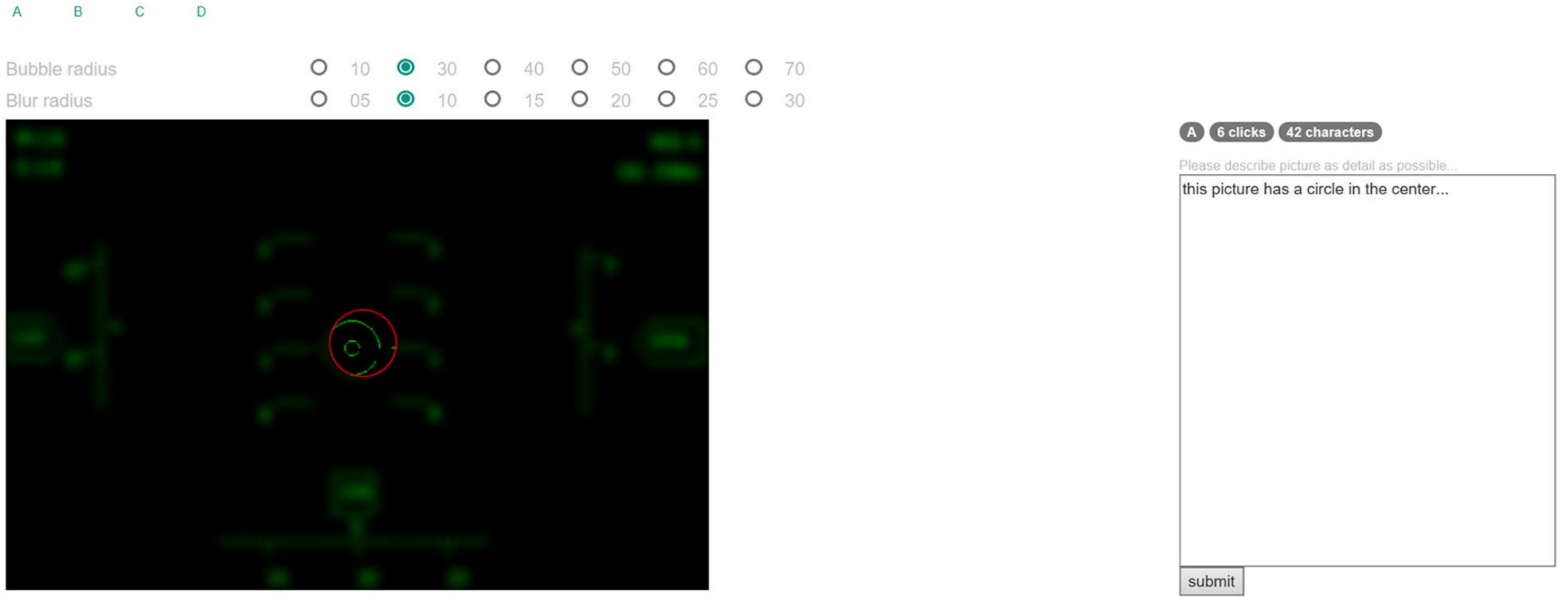
BubbleView experiment interface. The experiment requires users to reveal the picture by clicking (left), and describe the picture as thoroughly as possible during the exploration of the picture (right). The region included in the red circle means the subject has noticed this area

#### Data recording and preprocessing

We used JavaScript to write the BubbleView page (Fig. 3) and published it on the local server for receiving data. Data obtained from the BubbleView page is of two types: mouse click data (including the click position of the mouse and the number of mouse clicks) of the subject under different HMD interfaces, and text description of the current HMD interface. When the user clicked “submit” button, the data were sent to the local server and stored locally as a txt file. Our requirement for the data was that the subjects reveal the whole image as completely as possible while providing accurate and detailed textual descriptions. Therefore, we checked the mouse click range and analyzed the text description to delete some invalid data, and obtained 15 subjects’ valid data at last. Specifically, for the mouse click range, we visualized a binary mask image with the mouse click position as the center and a radius of 30 pixels (the bubble radius) to check if there are any unexplored areas. We filtered the text description one by one according to whether the description matched the interface information. For example, a circle will be seen if the picture is uncovered in the center. If the text description at this position does not have this key information, it will be considered invalid. We used Matlab to visualize the mouse click position and click times of 15 subjects under the four different HMD interfaces, and generated the visual importance heatmap of the corresponding interface [9].

### Statistics and analysis

When analyzing the experimental results, we compared the results of the first group and the second group, call it the first comparison group, and compared the results of the third group and the fourth group as the second comparison group. The statistical analyses of $2 \times 4$ repeated measures ANOVAs were used to analyze the amplitude and latency of target stimulus P2 response including factors of interfaces ([interface A vs. interface B] or [interface C vs. interface D]) and electrodes ([Fz, FCz, Cz, Pz]) [47]. We took 200ms to 275ms after the stimulus onset as the peak detection window of P2 component and calculated the average amplitude of P2 after stimulus onset from 205ms to 290ms [48].

**Fig. 4:**
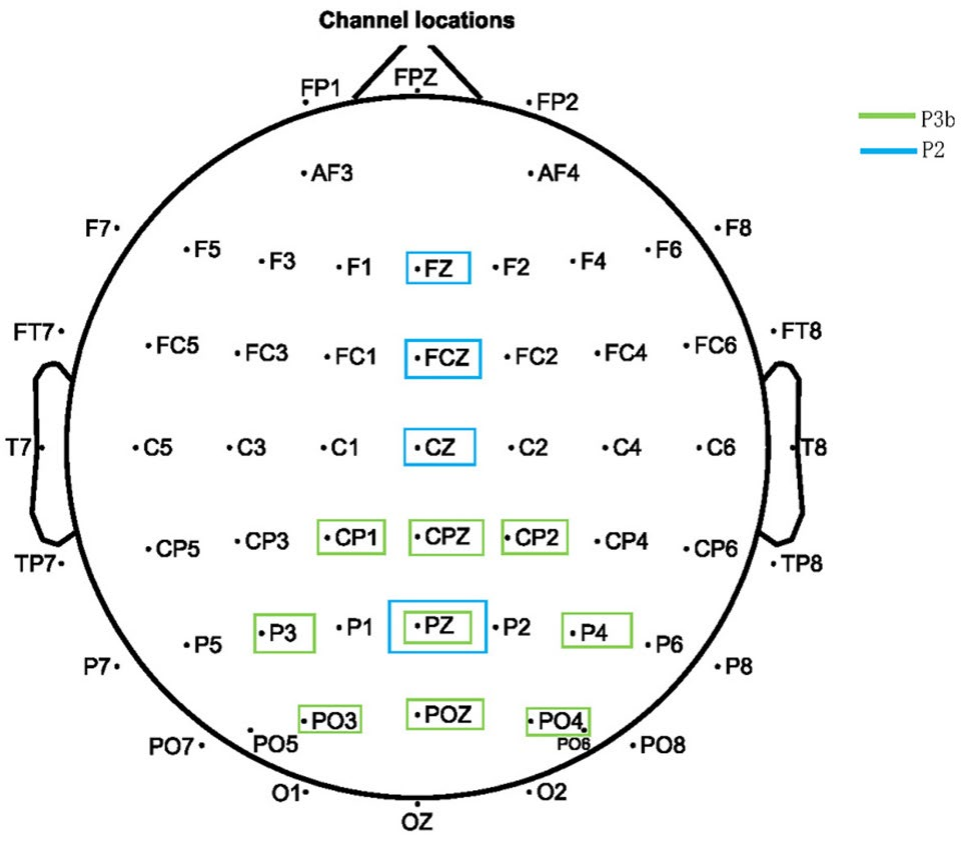
P2/P3 analysis electrodes. Electrodes that are used to analyze P2 component are included in blue box. Electrodes that are used to analyze P3b component are included in the green box

The statistical analyses of $2\times 3\times 3$ repeated measures ANOVAs were used to analyze the amplitude and latency of P3b response including factors of interfaces ([interface A vs. interface B] or [interface C vs. interface D]) and horizontal electrodes’ location (central parietal vs. parietal vs. occipital) and vertical electrodes’ location (left vs. central vs. right) [49]. CP1, CPz, and CP2 (Fig. 4) are included in the central parietal. P3, Pz, and P4 are included in the parietal lobe. PO3, POz, and PO4 are included in the occipital. P3, PO3, and CP1 are included on the left. CPz, Pz, and POz are included in central. CP2, P4, and PO4 are included on the right. We took the 300ms to 800ms after stimulus onset as the peak detection window of P3b component and calculated the average amplitude of P3b according to the 350ms to 800ms after stimulus onset [50].

We used the result of Mauchly’s test of sphericity to determine whether there are correlations between repeated measurement data, if so, the Greenhouse–Geisser will be used to correct the result. Paired sample *t*-test was used to analyze the significant difference in reaction time, accuracy and the mouse click times of subjects under different HMD interfaces. All statistical analysis operations were completed in IBM SPSS Statistics 21, and the statistical analysis threshold of $\alpha$ was set as ${p} < 0.05$.

**Table 2:** Data collected from two experiments under different HMD interfaces

Data type	Index	A	B	C	D
Behavior data	Reaction time(ms)	464.55 (94.41)	450.36 (87.65)	487.49 (74.42)	461.77 (77.90)
	Accuracy($\%$)	99.40	99.57	99.26	99.60
P3b	Latency(ms)	495.41 (6.63)	481.26(7.22)	514.38(7.53)	496.62(12.80)
	Amplitude($\mu \hbox {V}$)	2.12 (1.05)	8.84 (1.00)	7.08(0.69)	4.95(0.81)
P2	Latency(ms)	255 (1.73)	250 (6.13)	247 (6.66)	251(3.16)
	Amplitude($\mu \hbox {V}$)	– 0.71 (0.98)	1.00 (0.30)	– 0.09 (0.39)	– 0.24 (0.52)
BubbleView	Average clicks	79.80 (44.70)	88.93 (50.07)	101.67 (70.34)	86.60 (44.57)

(In parentheses is the standard deviation)

**Fig. 5:**
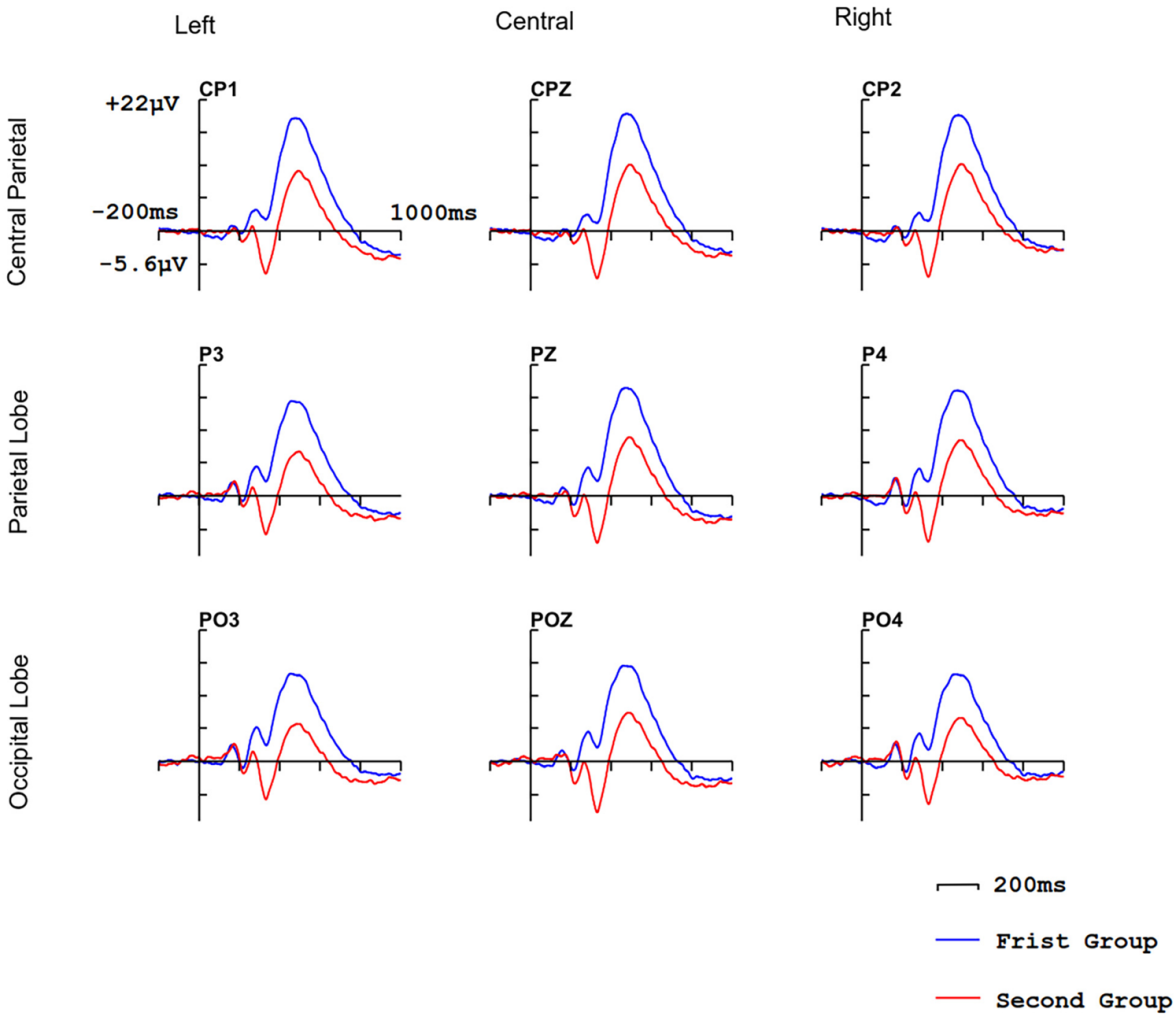
ERP waves on CP1, CPz, CP2, P3, Pz, P4, PO3, POz, PO4 (electrodes that are used to analyze P3b component) of the first comparison group (first group and second group)

**Fig. 6:**
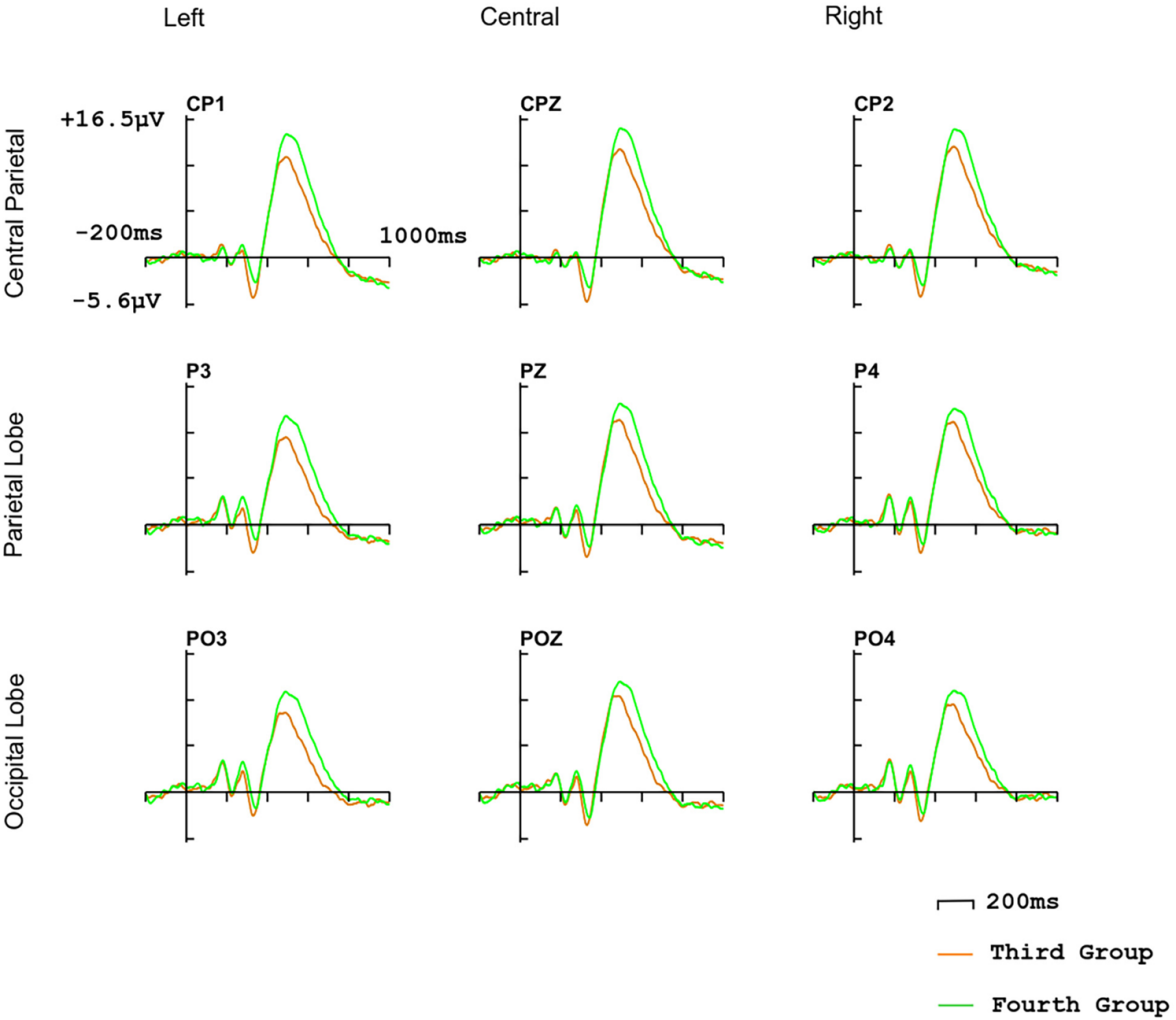
ERP waves on CP1, CPz, CP2, P3, Pz, P4, PO3, POz, PO4 (electrodes that are used to analyze P3b component) of the second comparison group (third group and fourth group)

## Result

### Behavior result

Average reaction time and accuracy under different interfaces are shown in Table 2. Response time under interface A is significantly slower than interface B (*t* = $-$3.303, *p* = 0.001). Response time under interface C is significantly slower than interface D. There is no significant difference in the two comparison groups on accuracy (the first comparison group: *t* = 1.157, *p* = 0.262; the second comparison group: *t* = 1.533, *p* = 0.143).

**Fig. 7:**
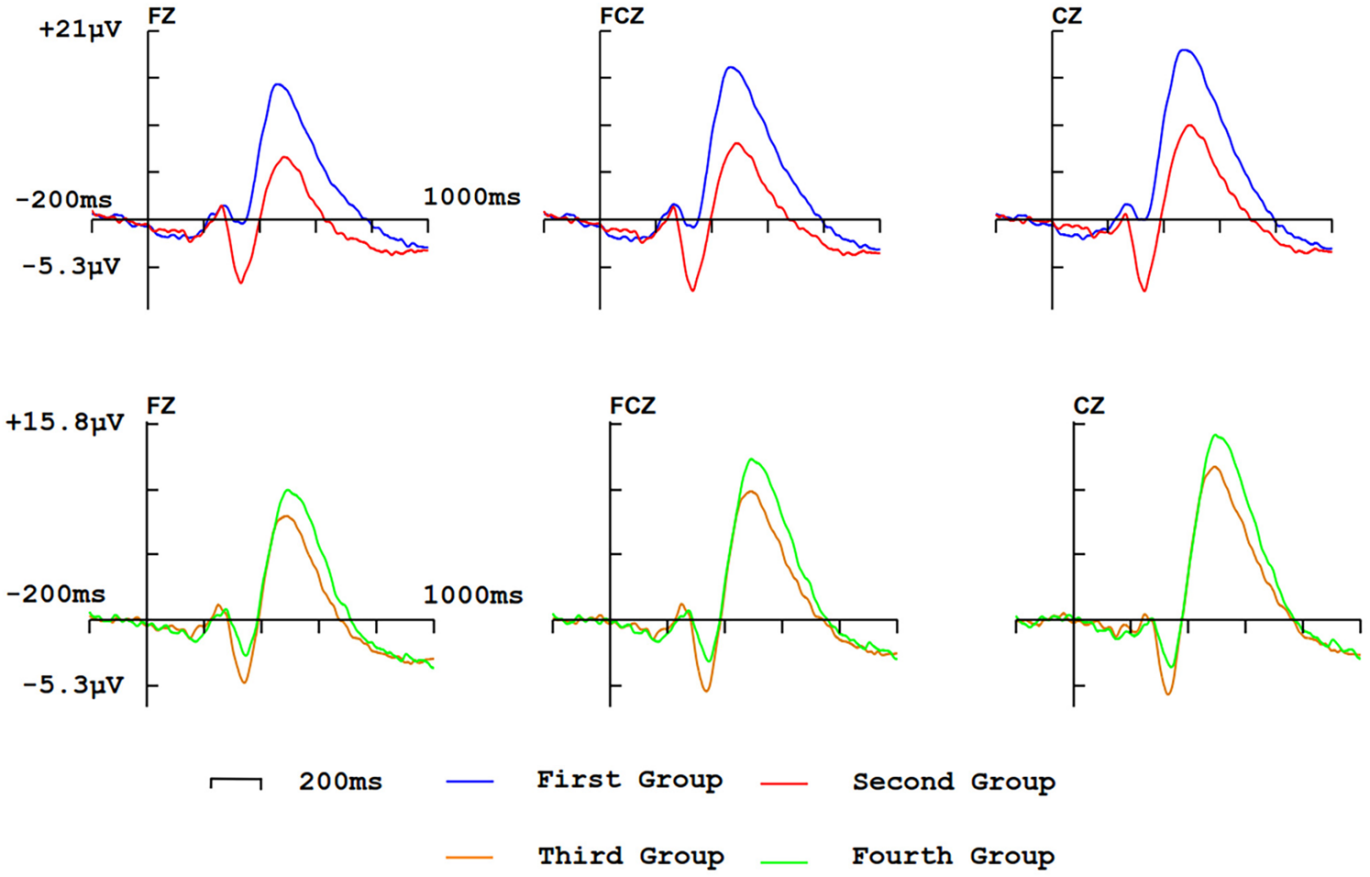
ERP waves on Fz, FCz, Cz (electrodes that are used to analyze P2 component) in each group

**Fig. 8:**
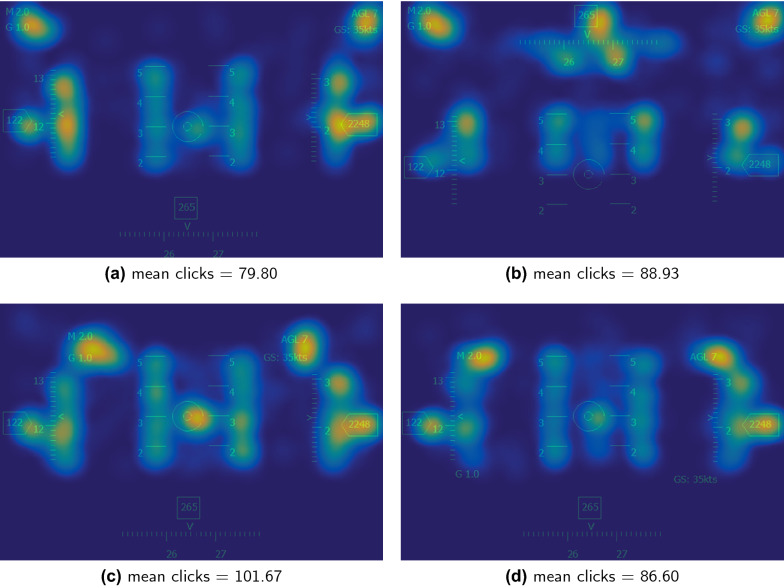
Mean clicks under different HMD interfaces. The color more bright, the more attention resources the participants allocate in this area

### ERP result

#### P3b

ERP waves elicited by target stimulus are shown in Figs. 5, 6. In the first comparison group, there is no significant difference on the latency of P3b (*p* = 0.071), P3b amplitude of interface B ($\hbox {amplitude} = 8.84\mu \hbox {V}$) is significantly higher than interface A ($\hbox {amplitude} = 2.12\mu \hbox {V}$), and the horizontal electrodes’ location has a significant impact on the P3b amplitude (${P} = 0.033$), the central parietal lobe ($\hbox {amplitude} = 6.58\mu \hbox {V}$) > parietal lobe ($\hbox {amplitude} = 5.47\mu \hbox {V}$) > occipital lobe ($\hbox {amplitude} = 4.79\mu \hbox {V}$), vertical electrodes’ location also has significantly influence on the amplitude (${P} = 0.045$), the central ($\hbox {amplitude} = 5.93\mu \hbox {V}$) > right ($\hbox {amplitude} = 5.84\mu \hbox {V}$) > left ($\hbox {amplitude} = 5.17\mu \hbox {V}$). In the second comparison group, there is no significant difference in the latency of P3b (${p} = 0.067$) and no significant difference in amplitude (${p} = 0.074$).

#### P2

ERP waves elicited by target stimulus are shown in Fig. 7. In the first comparison group, there is no significant difference in the latency of P2 (${p} = 0.374$), and the amplitude of P2 induced by interface B ($\hbox {amplitude} = 1.00\mu \hbox {V}$) is significantly higher than interface A ($\hbox {amplitude} = -0.71\mu \hbox {V}$). The interaction effect between the experimental group and the electrodes’ location is significant (${p} = 0.003$), but the effect of electrodes’ location on the amplitude is not significant (${p} = 0.309$). In the second comparison group, there is no significant difference in the latency (${p} = 0.472$) and the amplitude ($\hbox {p} = 0.472$) of P2. By calculating the correlation between the amplitude of P2 and P3b, it can be found that they have a positive correlation (Pearson correlation is 0.57, ${p} = 0.005$).

### BubbleView result

We use the mouse click data to generate the weight map and then superimpose the weight map and the original picture to generate the visual importance heatmap (Fig. 8). The color more bright, the more attention resources the participants allocate in this area. In the first comparison group, the mouse clicks of interface B are significantly more than interface A (${p} = 0.019$). The second comparison group has no significant difference (${p} = 0.174$).

## Discussion

In ERP, the visual P2 can be found in the context of the visual priming paradigm, which seeks to understand how prior information shapes future responses. In the visual priming paradigm experiment design, participants are briefly presented with an image or word, followed by a delay and a subsequent stimulus upon which participants must make a classification [51]. Researchers have used the visual search paradigm with stimulus arrays and found that target stimuli elicited larger anterior P2 components than standards. This evidence suggests that top-down information processing about feature classification affects processing at the visual perception stage. Thus, the P2 may index mechanisms for selective attention, feature detection (including color, orientation, shape, etc.), and other early stages of item encoding. Researchers found that 3D images induced P2 component with a larger amplitude than 2D images [46]. The amplitude of P2 component is sensitive to visual cognitive processing, which reflects the complexity of visual stimuli. Under the dual-task paradigm, the difficulty of the primary task is inversely proportional to the amplitude of P3b induced by the secondary task. When the primary task becomes more complex, there will allocate more attention resources or stimulus evaluation resources to the primary task, while more cognitive load and less attention resources will be allocated to the secondary task, and less P3b amplitude will be induced by the final secondary task [52]. Therefore, P3b amplitude directly reflects the difficulty of the task. The more reasonable the layout of the HMD interface, the subjects will invest the fewer attention resources or stimulus evaluation resources in cognitive recognition of the HMD interface. P2 component mainly reflects the evaluation process related to the physical attributes of stimulus images, which is a low-level evaluation process. In contrast, P3b component mainly reflects the evaluation process combining the overall layout rationality, which is a high-level evaluation process.

The amplitudes of P2 and P3b induced by interface B were significantly higher than that of interface A in Table 2. Interface B is asymmetrical [53] compared with interface A, and the icons are more crowded in interface B. So users spend more attention resources on cognitive judgment and memory, and have higher cognitive load on interface B. In the second comparison group, there is no significant difference in the amplitude of P2 and P3b. By comparison, the difference between interface C and interface D is a slight change in the layout of multiple small icons, meanwhile the amplitude of P3b is affected by the change and difference between target stimulus and standard stimulus [49], so there is no significant difference on the amplitude of P3b in the second comparison group. In addition, there is no significant difference in the latency of P2 and P3b for each interface, indicating that the evaluation of cognitive load by the latency of ERP in P2 and P3b is unreliable [49].

In the BubbleView experiments, we can find that the subjects spend less visual attention resources on the bottom half of the interface (Fig. 8), which is consistent with visual cognitive characteristics mentioned by [8]. There is a significant difference in the number of mouse clicks in the first comparison group. It shows that interface B has more information to process. There is no significant difference on the mouse clicks in the second comparison group. Also, no significant difference is found in the subjects’ accuracy under different HMD interfaces. This may be because the stimulus is presented for a long time (1000ms), and the interface is relatively simple.

ERP and BubbleView experiments both show that interface B has higher cognitive load than interface A. They also show that interface C and interface D have no significant difference on the amplitude, latency, and mouse clicks. The above analysis indicates that ERP and BubbleView have consistent evaluation results. However, the subjects’ reaction time on interface B is significantly faster than interface A (Table 2). The only difference between interface A and interface B is the location of the azimuth icon (interface A is below, and interface B is above). By analyzing the visual importance heatmap (Fig. 8), we find that the subjects’ attention resources are mainly concentrated on the top of the interface [8]. This indicates that although interface B has more information to process and a higher cognitive load, interface B is simpler to recognize and processes information more quickly because attention resources are primarily distributed in the upper part, resulting in a shorter reaction time than interface A. ERP results reflect the amount of attention resources input by subjects under different interfaces in order to understand interfaces, and also reflect different levels of cognitive load [32–34, 44–46], but fail to reflect the distribution of attention resources under different interfaces. Through BubbleView experiments, we explore the attention resources invested by subjects on the HMD interface to the allocation, the whole research process more completely and objectively reflects how different HMD interfaces are perceived and evaluated by subjects.

From the above analysis, the combination of ERP and BubbleView can be used to evaluate the cognitive load of digital interfaces. This method can reflect the entire cognitive process from input to allocation of attention resources, and the evaluation results of ERP and BubbleView for the cognitive load can be mutually verified. The BubbleView visual importance heatmap plays an essential role in explaining the significant differences in responses and the distribution of attention in the interface layout. Also, when designing an HMD interface, we must consider symmetry and simplicity to reduce cognitive load. In conclusion, combining ERP and BubbleView is an effective method to measure and evaluate the cognitive load of the interface.

## Conclusion

This paper proposes a new method combining ERP and BubbleView to evaluate and measure the cognitive load on digital interfaces. We designed ERP and BubbleView experiments for HMD interfaces with different layouts. By extracting the P3b and P2 components of the ERP, we analyzed the input of the subjects’ attentional resources on different interfaces and analyzed the participants’ cognitive load in different interfaces from two levels (low-level visual–physical attributes and high-level visual semantic information). Through the BubbleView experiment, we observed the allocation of subjects’ attention resources on the interface. Combining ERP and BubbleView, we can analyze the entire cognitive process of attention resources from input to allocation, thus evaluating the interface’s cognitive load. Experimental results show that HMD interfaces with a more symmetrical and concise layout have a less cognitive load, and subjects will pay more attention to the upper portion of the interface.

In future work, the interface evaluation method combining ERP and BubbleView can be applied not only to HMD interfaces, but also to other digital interfaces made of icon elements ordered according to predefined rules. The results of BubbleView can also be used to measure cognitive load, in particular circumstances without ERP experimental conditions.

## Data Availability

Not applicable.

## References

[1] Wang Y, Liu H (2014). Ergonomic review of airborne helmet aiming display system. Electron Opt Control.

[2] Wang Y (2013). Research on dynamic performance of airborne helmet aiming display system. Electron Opt Control.

[3] Wang H, Liu C, Wang Y (2012). Review of helmet display system. Electron Opt Control.

[4] Xia J, Miaomiao F, Zhang Y (2021) Based on visual cognition characteristic of helmet-mounted displays a summary of the interface design. 2021 26th International Conference on Automation and Computing (ICAC), p 1–6

[5] Murthy L, Mukhopadhyay A, Arjun S, Yelleti V, Thomas P, Mohan DB, Biswas P (2022). Eye-gaze-controlled HMDS and MFD for military aircraft. JATE.

[6] Goldberg JH, Kotval XP (1999). Computer interface evaluation using eye movements: methods and constructs. Int J Ind Ergon.

[7] Li WC et al (2020) Evaluating pilot′s perceived workload on interacting with augmented reality device in flight operations. In: Harris D, Li WC (eds) Engineering psychology and cognitive ergonomics. Cognition and Design. HCII 2020. Lecture Notes in Computer Science, vol 12187. Springer, Cham. 10.1007/978-3-030-49183-3_26

[8] Jia X, Xue C, Niu Y, Chen Y (2016) The design and evaluation methodologies of helmet-mounted display symbology. In: 2016 23rd international conference on mechatronics and machine vision in practice (M2VIP), pp 1–4

[9] Kim NW, Bylinskii Z, Borkin MA, Gajos KZ, Oliva A, Durand F, Pfister H (2017). Bubbleview: an alternative to eye-tracking for crowdsourcing image importance. ACM Trans Comput Hum Interact.

[10] Fowler B (1994). P300 as a measure of workload during a simulated aircraft landing task. Hum Factors.

[11] Kramer AF, Sirevaag EJ, Braune R (1987). A psychophysiological assessment of operator workload during simulated flight missions. Hum Factors.

[12] Sweller J (1988). Cognitive load during problem solving: effects on learning. Cogn Sci.

[13] Jing L, Shulan Y, Wei L (2017). Cognitive characteristic evaluation of CNC interface layout based on eye-tracking. J Comput Aided Des Comput Graph.

[14] Giraudet L, Imbert J, Berenger M, Tremblay S, Causse M (2016). Erratum to the neuroergonomic evaluation of human machine interface design in air traffic control using behavioral and EEG/ERP measures. Behav Brain Res..

[15] Wei Z, Wanyan X, Zhuang D (2014). Measurement and evaluation of mental workload for aircraft cockpit display interface. J Beijing Univ Aeronaut Astronaut.

[16] Johansen SA, Nørgaard M, Soerensen JR (2008) Can eye tracking boost usability evaluation of computer games? In: CHI 2008: evaluating user experiences in games

[17] Wu Y, Cheng J, Kang X (2016) Study of smart watch interface usability evaluation based on eye-tracking. In: Marcus A (ed) Design, user experience, and usability: technological contexts. DUXU 2016. Lecture Notes in Computer Science, vol 9748. Springer, Cham. 10.1007/978-3-319-40406-6_10

[18] Açik A, Erol D, Akgün G, Yantaç A, Aydin Ç (2016). Evaluation of a surgical interface for robotic cryoablation task using an eye-tracking system. Int J Hum Comput Stud.

[19] Zhang X, Sugano Y, Fritz M, Bulling A (2015) Appearance-based gaze estimation in the wild. 2015 IEEE Conference on Computer Vision and Pattern Recognition (CVPR), pp 4511–4520

[20] Huang Q, Veeraraghavan A, Sabharwal A (2017). Tabletgaze: dataset and analysis for unconstrained appearance-based gaze estimation in mobile tablets. Mach Vis Appl.

[21] Krafka K, Khosla A, Kellnhofer P, Kannan H, Bhandarkar SM, Matusik W, Torralba A (2016) Eye tracking for everyone. 2016 IEEE Conference on Computer Vision and Pattern Recognition (CVPR), pp 2176–2184

[22] Rodden K, Fu X, Aula A, Spiro I (2008) Eye-mouse coordination patterns on web search results pages. In: CHI'08 extended abstracts on human factors in computing systems, pp 2997–3002

[23] Guo Q, Agichtein E (2010) Towards predicting web searcher gaze position from mouse movements. In: CHI'10 extended abstracts on human factors in computing systems, pp 3601–3606

[24] Jansen AR, Blackwell AF, Marriott K (2003). A tool for tracking visual attention: The restricted focus viewer. Behav Res Methods Instrum Comput.

[25] Gomez SR, Jianu R, Cabeen RP, Guo H, Laidlaw DH (2017). Fauxvea: Crowdsourcing gaze location estimates for visualization analysis tasks. IEEE Trans Visual Comput Graphics.

[26] Kim NW, Bylinskii Z, Borkin MA, Oliva A, Gajos KZ, Pfister H (2015) A crowdsourced alternative to eye-tracking for visualization understanding. Proceedings of the 33rd Annual ACM Conference Extended Abstracts on Human Factors in Computing Systems

[27] Lyudvichenko V, Vatolin DS (2019) Predicting video saliency using crowdsourced mouse-tracking data. arXiv:abs/1907.00480

[28] Anwyl-Irvine AL, Armstrong T, Dalmaijer ES (2021). Mouseview.js: Reliable and valid attention tracking in web-based experiments using a cursor-directed aperture. Behav Res Methods.

[29] Luck SJ (2014). An introduction to the event-related potential technique.

[30] Hunter CR (2020). Tracking cognitive spare capacity during speech perception with EEG/ERP: effects of cognitive load and sentence predictability. Ear Hear.

[31] Swerdloff MM, Hargrove LJ (2020) Quantifying cognitive load using EEG during ambulation and postural tasks. 2020 42nd Annual International Conference of the IEEE Engineering in Medicine & Biology Society (EMBC), pp 2849–2852 10.1109/EMBC44109.2020.9176264 33018600

[32] Allison BZ, Polich J (2008). Workload assessment of computer gaming using a single-stimulus event-related potential paradigm. Biol Psychol.

[33] Miller M, Rietschel JC, Mcdonald C, Hatfield B (2011). A novel approach to the physiological measurement of mental workload. Int J Psychophysiol.

[34] Ullsperger P, Freude G, Erdmann U (2001). Auditory probe sensitivity to mental workload changes - an event-related potential study. Int J Psychophysiol.

[35] Squires N, Squires K, Hillyard S (1975). Two varieties of long-latency positive waves evoked by unpredictable auditory stimuli in man. Electroencephalogr Clin Neurophysiol.

[36] Santangelo V, Belardinelli MO, Spence C (2007). The suppression of reflexive visual and auditory orienting when attention is otherwise engaged. J Exp Psychol Hum Percept Perform.

[37] Scott S, Albert C, Salvador SF (2006). Manipulating inattentional blindness within and across sensory modalities. Q J Exp Psychol.

[38] Lavie N (1995). Perceptual load as a necessary condition for selective attention. J Exp Psychol Hum Percept Perform.

[39] Lavie N, Hirst A, de Fockert JD, Viding E (2004). Load theory of selective attention and cognitive control. J Exp Psychol Gen.

[40] Berti S, Schröger E (2003). Working memory controls involuntary attention switching: evidence from an auditory distraction paradigm. Eur J Neurosci.

[41] Munka L, Berti S (2006). Examining task-dependencies of different attentional processes as reflected in the p3a and reorienting negativity components of the human event-related brain potential. Neurosci Lett.

[42] Stefan B (2013). The role of auditory transient and deviance processing in distraction of task performance: a combined behavioral and event-related brain potential study. Front Hum Neurosci.

[43] Potts G, Tucker D (2001). Frontal evaluation and posterior representation in target detection. Brain Res Cogn Brain Res.

[44] Tong Y, Melara RD, Rao A (2009). P2 enhancement from auditory discrimination training is associated with improved reaction times. Brain Res.

[45] Ying L, Fu S, Qian X, Sun X (2011). Effects of mental workload on long-latency auditory-evoked-potential, salivary cortisol, and immunoglobulin A. Neurosci Lett.

[46] Shu O, Kuroiwa Y, Otsuka S, Baba Y, Wang C, Li M, Mizuki N, Ueda N, Koyano S, Suzuki Y (2010). P1 and P2 components of human visual evoked potentials are modulated by depth perception of 3-dimensional images. Clin Neurophysiol.

[47] Jaquess KJ, Gentili R, Lo L-C, Oh H, Zhang J, Rietschel JC, Miller M, Tan YY, Hatfield B (2017). Empirical evidence for the relationship between cognitive workload and attentional reserve. Int J Psychophysiol.

[48] Raz S, Dan O, Zysberg L (2014). Neural correlates of emotional intelligence in a visual emotional oddball task: An erp study. Brain Cogn.

[49] Niu Y, Xie Y, Xue C, Wang H, Tang W, Guo Q, Jin T (2018). Investigation on the neurophysiological correlates of similarity cognition of digital interface color and layout. Adv Mech Eng.

[50] Coleman JR, Turrill J, Hopman R, Cooper JM, Strayer D (2017) Assessing cognitive distraction using event related potentials. In: Driving Assessment Conference

[51] Luck S, Hillyard AS (1994). Electrophysiological correlates of feature analysis during visual search. Psychophysiology.

[52] Kok A (2010). On the utility of p3 amplitude as a measure of processing capacity. Psychophysiology.

[53] Bauerly M, Liu Y (2005). Development and validation of a symmetry metric for interface aesthetics. Proc Hum Factors Ergon Soc Annu Meet.

